# Supplementary material for: Ultrastructural analysis in yeast reveals a meiosis-specific actin-containing nuclear bundle
Source: Commun Biol. 2021 Aug 25;4:1009. doi: 10.1038/s42003-021-02545-9 (PMC8387383; doi:10.1038/s42003-021-02545-9)
Supplement: Supplementary file 2 — Supplementary Information [file 42003_2021_2545_MOESM2_ESM.pdf]

**Supplementary Information**

# Ultrastructural analysis in yeast reveals a meiosis-specific actin-containing nuclear bundle

**Tomoko Takagi, Masako Osumi and Akira Shinohara**

**Supplementary Figure 1. EM images of nuclear bundles for measurement of diameter and inter-filament distance**

**a-f.** A representative image of cross-sectioned bundles is shown (a) with a magnified view (b), which is converted to a noise-removed binary image (c). An elliptical fitting image of each filament was taken (d). A short axis (red) was measured as a diameter (e). Distances between centers of each filament were measured as an inter-filament distance (bule line, f).

**g.** Representative images of nuclear bundles with possible branches. Two representative lines (red in g') were drawn for the measurement for an angle. Bar indicates100 nm (a) and 200 nm (g).

**Supplementary Figure 2. Properties of cytoplasmic bundles**

**a.** Kinetics of the formation of the bundles in nuclei (red) and cytoplasm (green) at different times of meiosis. A number of sections containing bundles were counted and percentages of bundle-positive nuclei and -cytoplasm section as well as microtubule-positive nucleus section are shown.

**b.** Distribution of a diameter of the filament in cytoplasmic bundles in nuclei at 4 h in meiosis. The diameter of filaments in a cross-section were measured shown in Supplementary Figure 1 and ranked every 2 nm. The number of each rank in two independent images is shown in different colors ($n$=21, 9).

**c.** Distribution of a distance between two adjacent filaments in cytoplasm at 4 h in meiosis. The distance between two adjacent filaments in a cross-section was measured shown in in Supplementary Figure 1 ($n$=20) and ranked every 2 nm.

**Supplementary Figure 3. EM images of chemically fixed meiotic yeast cells in mid-prophase I**

Transmission electron microscopy (TEM) images of a yeast diploid cell after incubation with SPM for 0 h (a, b), 4 h (c, d, e), and 6 h (f, g). The specimens were fixed with chemicals (glutaraldehyde and $OsO_4$) and sectioned. Magnified images with a bundle in nucleus (d, g) are shown. (e) A magnified image of a cytoplasm bundle of another 4h cell. Bars indicate 1 μm (a, c, f) and 200 nm (b, d, e, g). M, mitochondrion; N, nucleus; V, vacuole. Arrows indicate nuclear bundles.

**Supplementary Figure 4. Serially sectioned EM images of a chemically fixed**

**yeast cell in mid-prophase I**

Serial sectioned TEM images of an identical cell at 4 h, cells were fixed with chemicals (glutaraldehyde and $OsO_4$). Bar indicates 500 nm. Arrows indicate nuclear bundles.

**Supplementary Figure 5. Kinetics of bundle formation in *spo11* cells**

Kinetics of the formation of bundles in nuclei (red) and cytoplasm (green) as well as nuclear microtubules (blue) at different times of meiosis of the *spo11-Y135F* mutant (HSY185/186). A number of sections containing bundles was counted and percentages of bundle-positive nuclei and cytoplasm sections are shown.

**Supplementary Figure 6. EM analysis of SC and filasomes**

**a.** Representative TEM images with nuclear bundles with a possible synaptonemal complex-like structure (SC?) at 5 h is shown in (a). M, mitochondrion; F, filasome; N, nucleus; Nu, nucleolus. Bar indicates 200 nm.

**b-f.** Images of a filasome in a yeast diploid cell are shown. Filasomes are less dense structures with a vesicle in the center, devoid of ribosomes in cytoplasm. An average density of ribosomes per a filasome is 3.3±0.41 ribosomes pixel$^{-2}$ ($n$=13) while that in cytoplasm is 22.4±0.96 ribosomes pixel$^{-2}$ ($n$=26). Bar indicates 100 nm.

**Supplementary Figure 7. Immuno-gold labeling using anti-actin antibody**

The specimens were prepared with freezing-fixation. Immuno-gold labeling using anti-actin antibody was carried out as described in Methods. A representative image (a) containing actin bundles in nucleus (b) and in cytoplasm (c) in a cell at 4 h are shown with magnified images (b, c). Magnified images of nuclear bundle (d) and cytoplasmic bundle (e) containing gold particles of another cells. The positions of gold particles are shown in arrowheads (b-e). Bar indicates 200 nm (a) and 100 nm (b-e).

**Supplementary Figure 8. Immuno-gold labeling using anti-actin antibodies at nuclei**

Representative immuno-gold electron microscopy images of a yeast diploid cell at 4 h. The specimens were prepared with freezing-fixation. Immuno-gold labeling using anti-actin antibodies (MAB1501R) (a) and (MAB8172) (b-d) were shown at bundles and high-density regions. The positions of gold particles are shown in

arrowheads (a-d). Bar indicates 200 nm. CW, cell wall; L, Lipid body; M, mitochondrion; N, nucleus; V, vacuole.

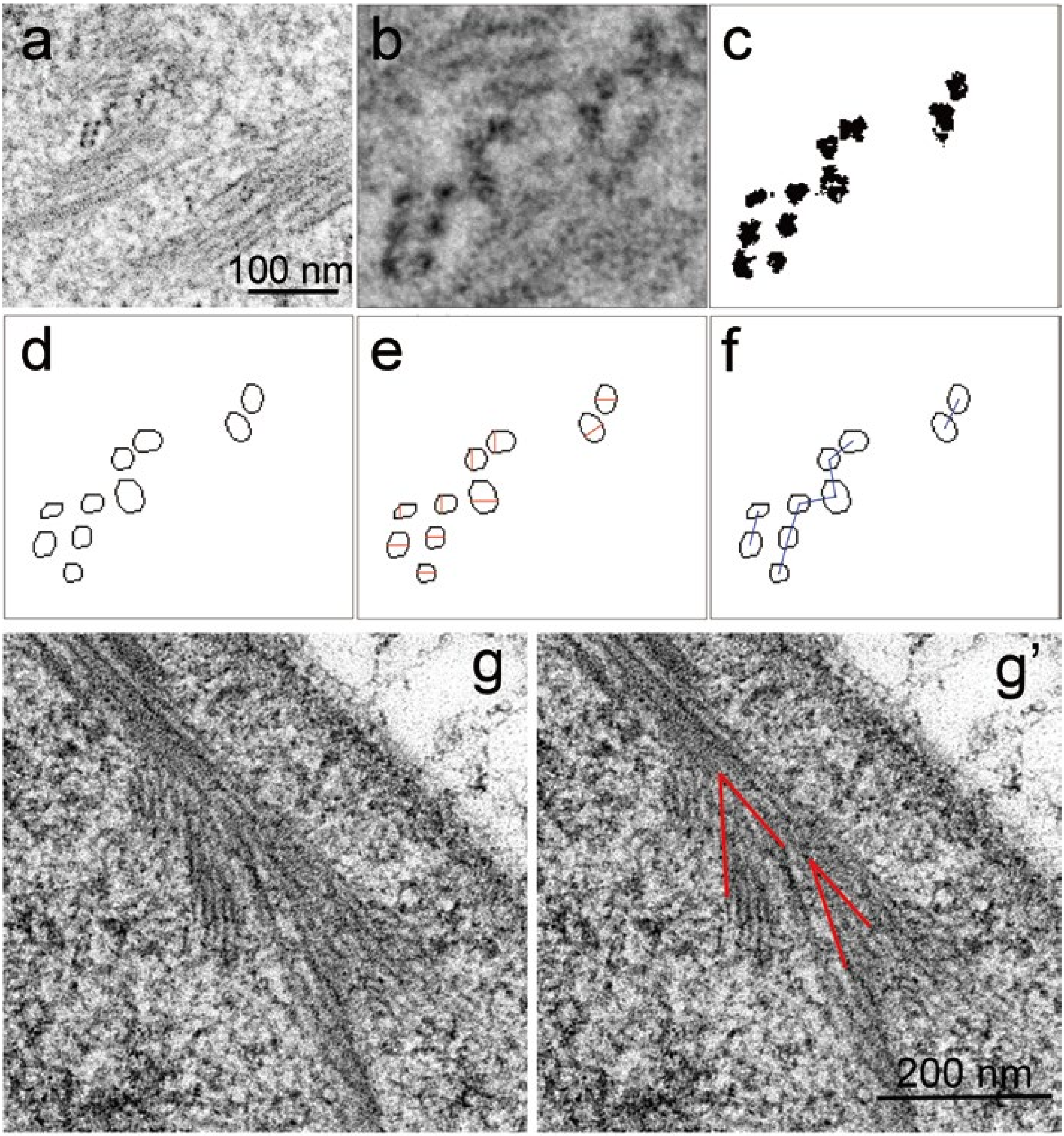


Supplementary Figure 1 Takagi *et al*.

# Supplementary Figure 2. Takagi

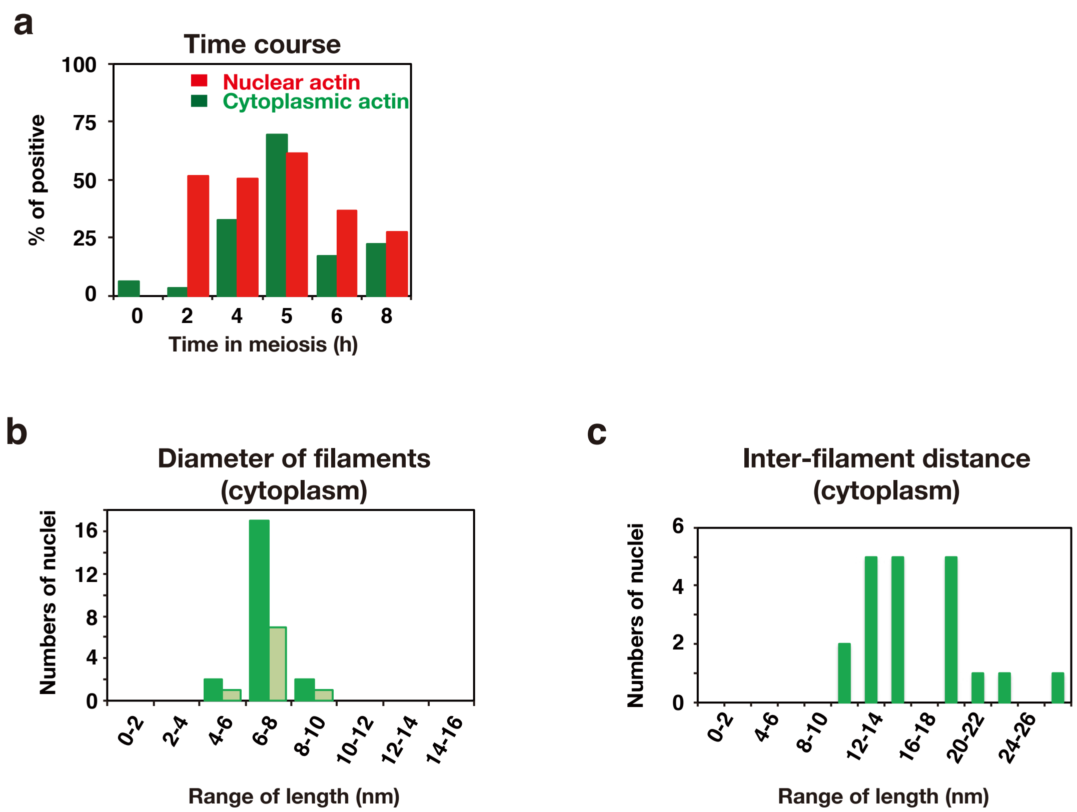

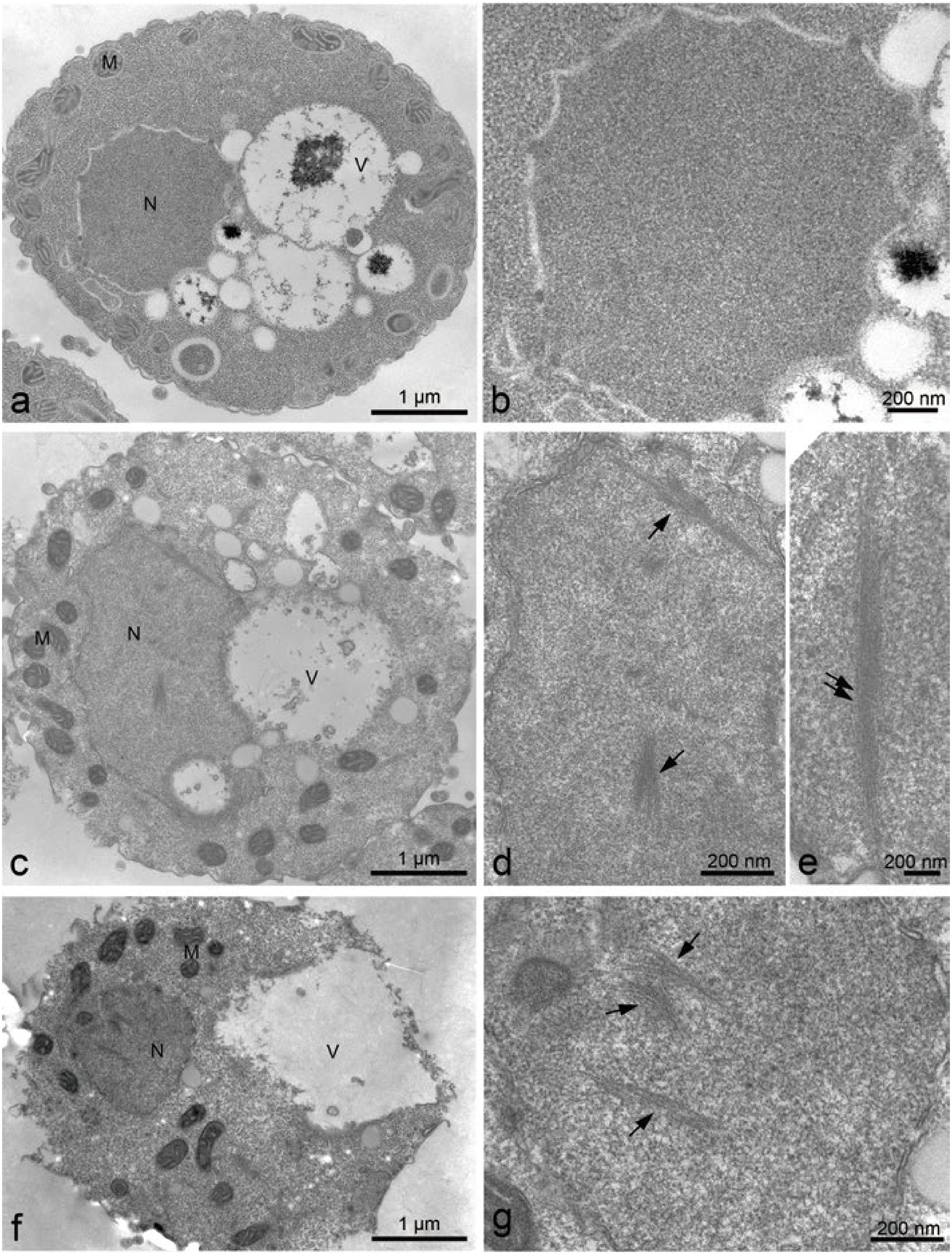


Supplementary Figure 3 Takagi *et al*.

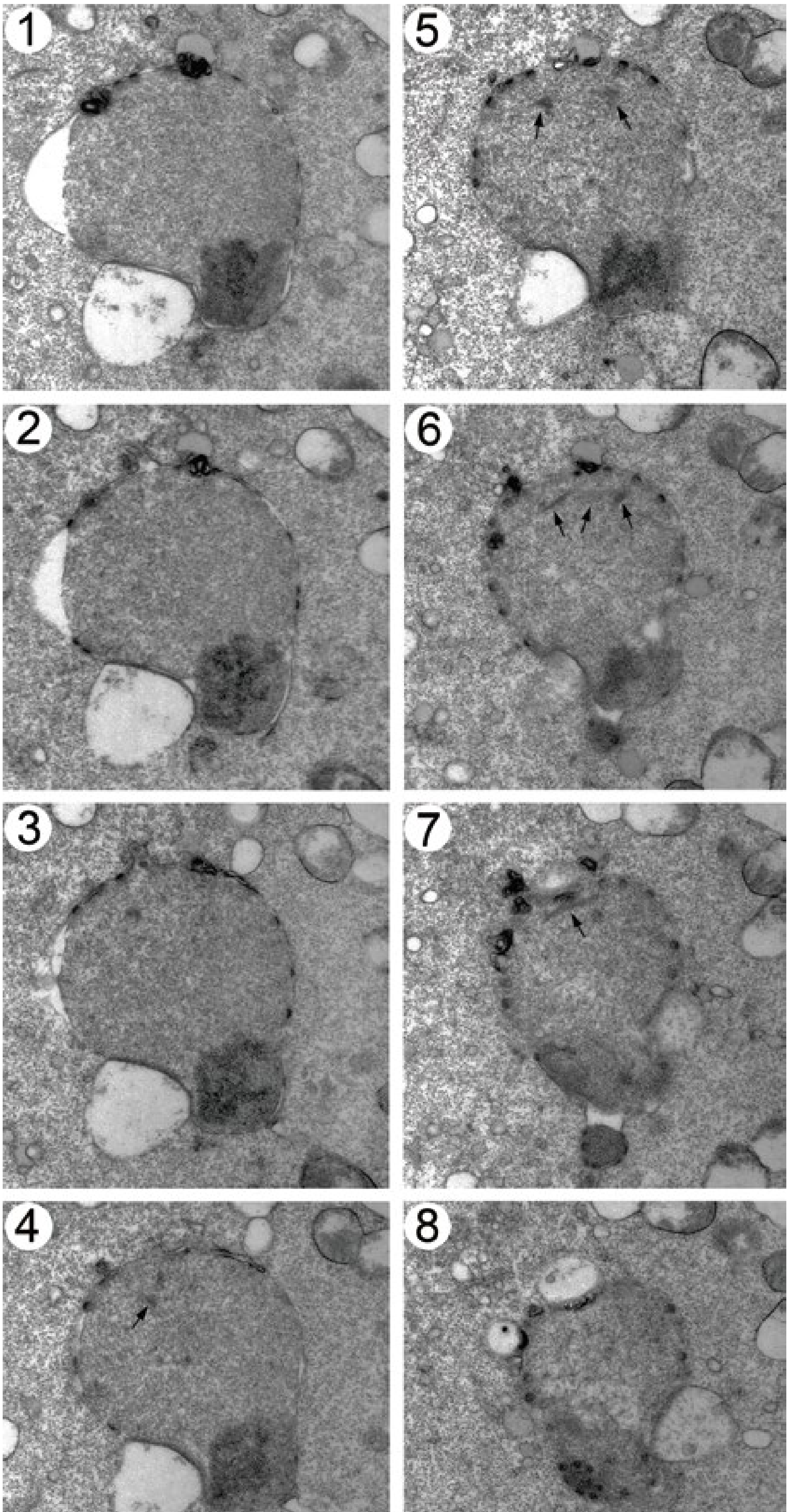


Supplementary Figure 4 Takagi *et al*.

# Supplementary Figure 5. Takagi

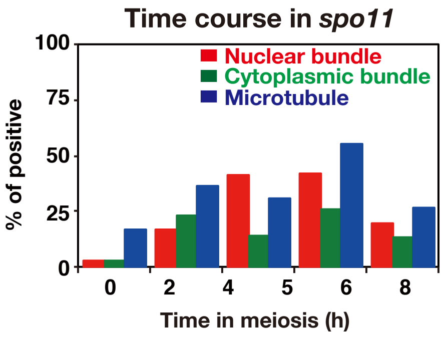

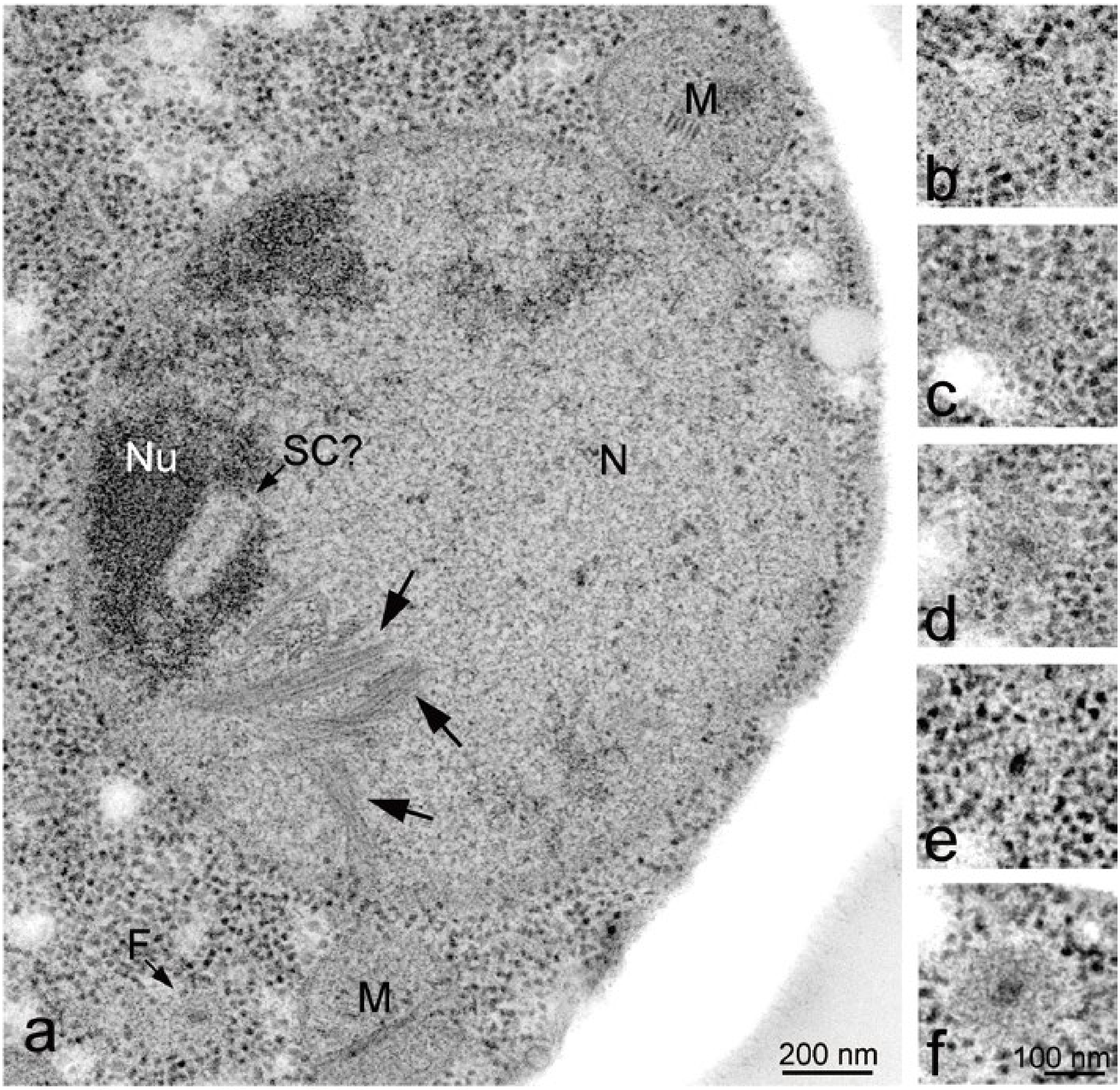


Supplementary Figure 6 Takagi *et al*.

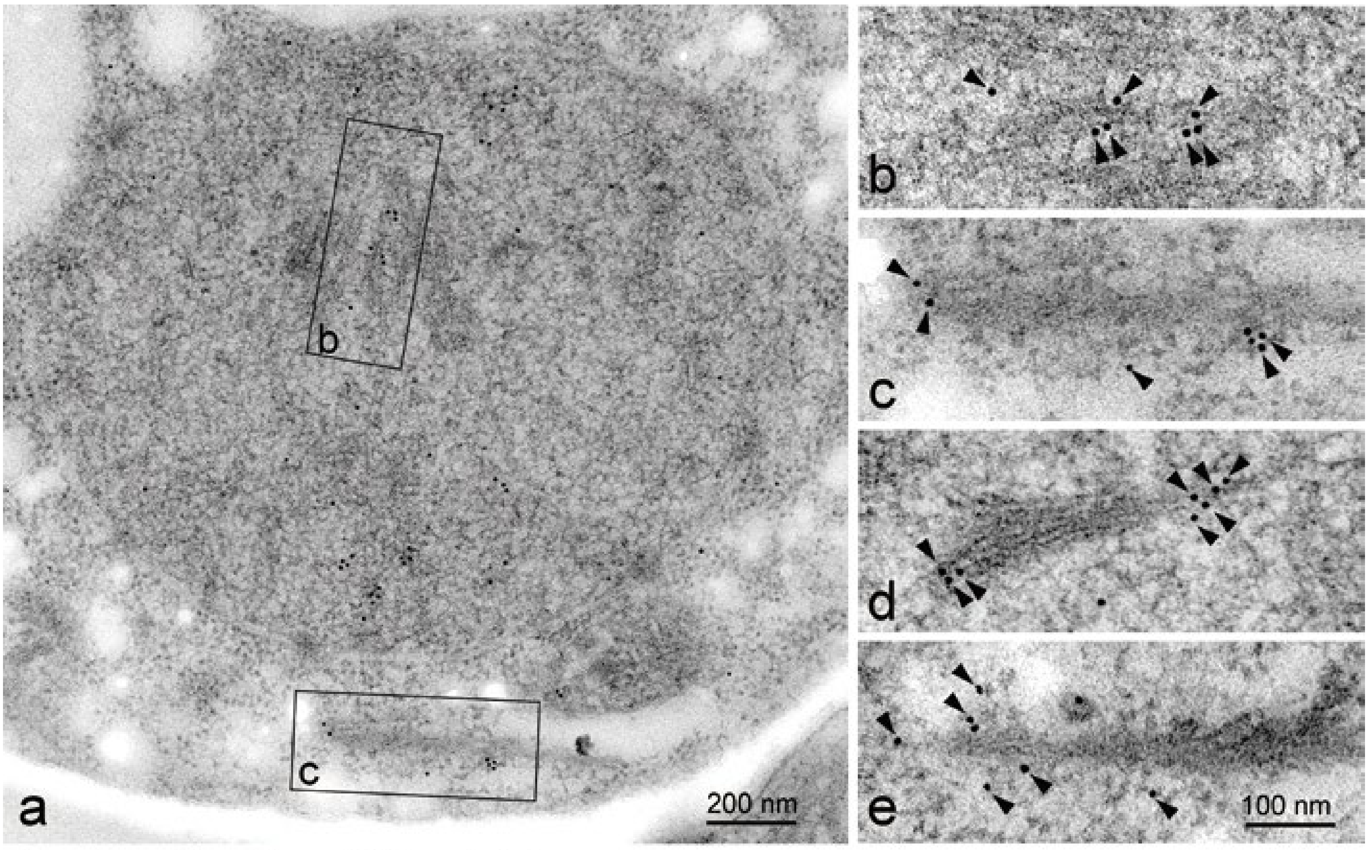


Supplementary Figure 7 Takagi *et al*.

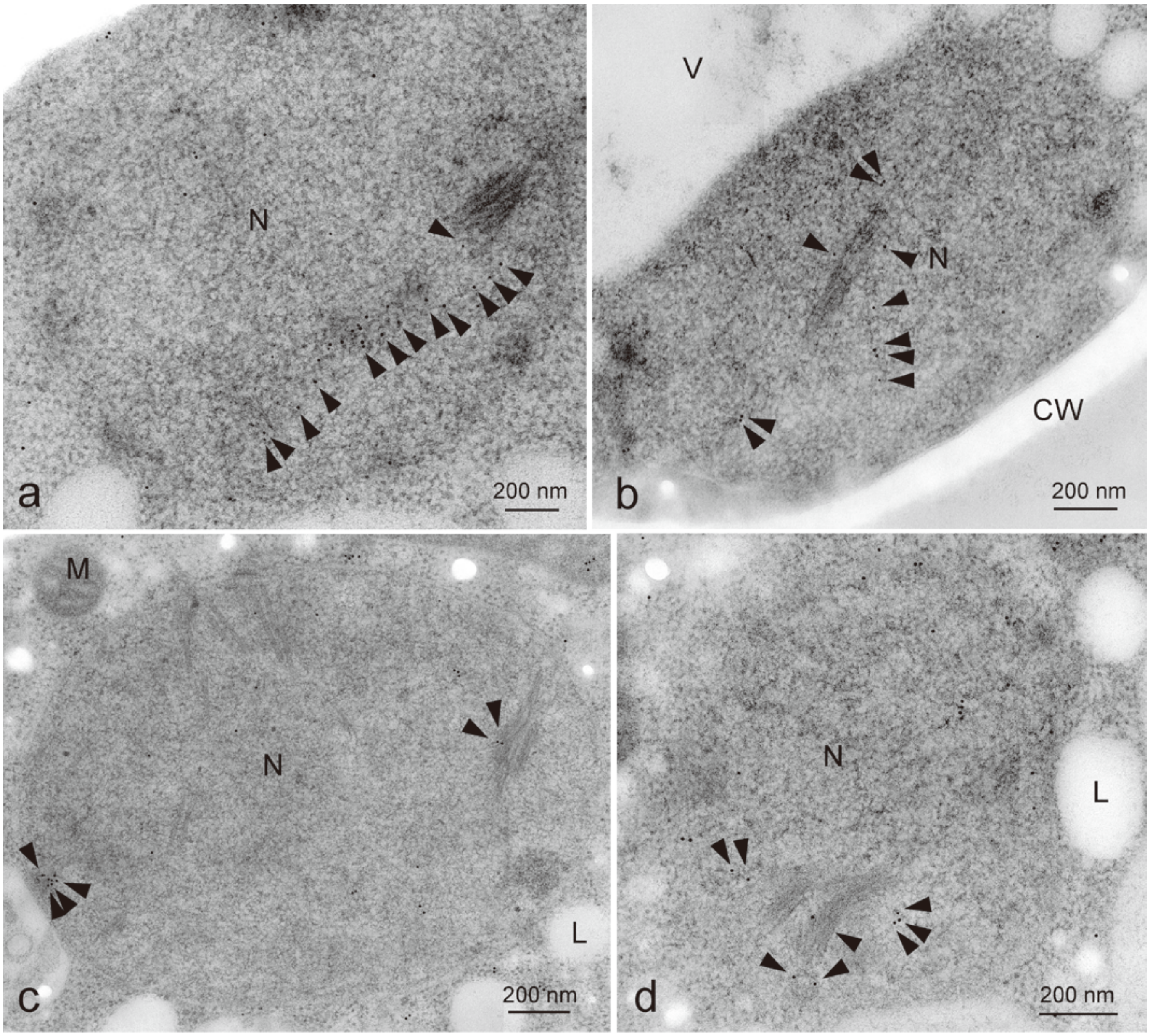


Supplementary Figure 8 Takagi *et al*.
