# Supplementary material for: Ultrastructural analysis in yeast reveals a meiosis-specific actin-containing nuclear bundle
Source: Commun Biol. 2021 Aug 25;4:1009. doi: 10.1038/s42003-021-02545-9 (PMC8387383; doi:10.1038/s42003-021-02545-9)
Supplement: Supplementary file 3 — Description of Supplementary Files [file 42003_2021_2545_MOESM3_ESM.docx]

**Description of Additional Supplementary Files**

**File name:** Supplementary Data 1

**Description:** Source data for Fig. 4 and Supplementary Figures 2 and 5.
